# Over-expression of Eph and ephrin genes in advanced ovarian cancer: ephrin gene expression correlates with shortened survival

**DOI:** 10.1186/1471-2407-6-144

**Published:** 2006-06-01

**Authors:** Nirmitha I Herath, Mark D Spanevello, Sabe Sabesan, Tanya Newton, Margaret Cummings, Shannon Duffy, Douglas Lincoln, Glen Boyle, Peter G Parsons, Andrew W Boyd

**Affiliations:** 1 Leukaemia Foundation Research Unit, University of Queensland, Australia; 2 Melanoma Genomics, University of Queensland, Australia; 3 Cancer and Population Studies, Queensland Institute of Medical Research, University of Queensland, Australia; 4 Faculty of Health Sciences, University of Queensland, Australia

## Abstract

**Background:**

Increased expression of Eph receptor tyrosine kinases and their ephrin ligands has been implicated in tumor progression in a number of malignancies. This report describes aberrant expression of these genes in ovarian cancer, the commonest cause of death amongst gynaecological malignancies.

**Methods:**

Eph and ephrin expression was determined using quantitative real time RT-PCR. Correlation of gene expression was measured using Spearman's rho statistic. Survival was analysed using log-rank analysis and (was visualised by) Kaplan-Meier survival curves.

**Results:**

Greater than 10 fold over-expression of EphA1 and a more modest over-expression of EphA2 were observed in partially overlapping subsets of tumors. Over-expression of EphA1 strongly correlated (r = 0.801; p < 0.01) with the high affinity ligand ephrin A1. A similar trend was observed between EphA2 and ephrin A1 (r = 0.387; p = 0.06). A striking correlation of both ephrin A1 and ephrin A5 expression with poor survival (r = -0.470; p = 0.02 and r = -0.562; p < 0.01) was observed. Intriguingly, there was no correlation between survival and other clinical parameters or Eph expression.

**Conclusion:**

These data imply that increased levels of ephrins A1 and A5 in the presence of high expression of Ephs A1 and A2 lead to a more aggressive tumor phenotype. The known functions of Eph/ephrin signalling in cell de-adhesion and movement may explain the observed correlation of ephrin expression with poor prognosis.

## Background

The sixteen vertebrate Eph receptors form the largest subfamily of receptor tyrosine kinase (RTK) proteins. Activation and signalling by these receptors is mediated by interaction with nine cell-surface counter receptors known as ephrins. The ephrins are subdivided into an A group which is glycosylphosphatidyl-inositol (GPI) anchored and a B group of type I trans-membrane proteins [1]. Eph proteins are also classified into A and B groups depending on structural features and preferential binding to either A or B type ephrins [2]. Eph and ephrin proteins have important roles in facilitating de-adhesion and cell movement, thereby playing critical roles in many developmental processes [1,3,4]. Dysregulation of cell adhesion and cell motility mechanisms have emerged as key elements in tumor progression and metastasis and it is notable that a large body of evidence details re-expression of both Eph and ephrin proteins at high levels in malignancies including melanoma and colon, gastric, breast, endometrial, and lung carcinomas [1,5].

EphB and ephrin B proteins have been implicated in both normal and malignant epithelial tissues. EphB2 and EphB3/ephrin B signalling regulates cell sorting in the mature gut epithelium [6], counter gradients of EphB and ephrin-B proteins regulating cell migration through contact-mediated cell repulsion [7]. Over-expression of EphB2, EphB4 and ephrin B1 is described in gastric, colon and breast cancers [8-12].

The EphA/ephrin A system is also expressed in normal and neoplastic epithelial tissues. Human EphA1 protein was isolated from the hepatocellular carcinoma cell line ETL-1 [13]. EphA1 is expressed in normal epithelial organs [14]. Over-expression of EphA1 has been described in prostate cancer [15], gastric cancers [16] and a subset of colon, lung, liver and mammary carcinomas [13,17]. EphA2 over-expression has been found in oesophageal, breast and prostate cancers [18,19]. In non-small cell lung cancer, high levels of EphA2 predicted metastasis [20]. Ephrin A1 is the high affinity ligand for both EphA1 [14] and EphA2. A study of EphA2 and ephrin A1 in the CaCo2 colon cancer line suggests that their interaction may be of importance in colon epithelial structure and function [21].

We describe the expression of Eph and ephrin genes in a series of ovarian cancers and non-malignant tissues using quantitative real time RT-PCR. We show that elements of both EphA/ephrin A and EphB/ephrin B signalling systems are over-expressed. A significant proportion of ovarian tumors showed a > 5-fold increase in expression of some Eph and/or ephrin proteins compared with non-malignant tissues. EphA1 and EphA2 over-expression was correlated with ephrin A1 suggesting that their interaction may have a role in ovarian cancer progression. Intriguingly, over-expression of ephrin A1 and ephrin A5 mRNA correlated with shortened survival whereas EphA1 and EphA2 expression did not. The potential consequences of this on ovarian cancer cell biology and clinical behaviour are discussed.

## Methods

### Patient samples

Cancer specimens from twenty-four patients with advanced ovarian cancer or primary peritoneal carcinoma (PPC) were obtained after de-bulking surgery at the Royal Brisbane Hospital (Queensland, Australia) after obtaining informed consent and approval by the ethics committees of the Queensland Institute of Medical Research, the University of Queensland and the Royal Brisbane Hospital. These specimens were reviewed by Dr M Cummings, Pathologist at the Royal Brisbane Hospital. The patient's progress was monitored through follow-up visits with their oncologists and CA 125 levels. Two normal ovaries, one benign ovarian adenoma and one ovarian endometriotic cyst were tested as non-malignant controls.

### RNA extraction and DNase digestion

Total RNA was isolated using the Qiagen RNeasy ^® ^Midi Kit (Qiagen Pty Ltd, Australia), according to the manufacturer's instructions. The initial part of the protocol was modified so that approximately 200 mg of tissue was crushed with a mortar and pestle in liquid nitrogen. RLT buffer containing 10μl/mlβ-mercaptoethanol (βME) was added and ground into the powdered tissue. Tissue was transferred to a conical tube and homogenized with a rotor/stator homogeniser (Polytron PT 1200, Kinematica, Switzerland) for 2 × 30 second bursts. Samples were digested with proteinase K (Qiagen Pty Ltd, Australia) and extraction continued as recommended by the manufacturer.

Prior to cDNA synthesis, the optical density of all RNA samples was measured and RNA integrity was assessed by gel electrophoresis. These samples were then subjected to DNase I treatment using RQ1 RNase-free DNase I (Promega Pty Ltd, Australia) following manufacturer's instructions.

### cDNA synthesis

First strand cDNA was synthesized by reverse transcription using Superscript III Reverse Transcriptase (Invitrogen Pty Ltd, Australia). Briefly, DNase I-digested RNA was incubated with 1.5 μl of 500 μg/ml oligo-dT_30, _1.5 μl of 10 mM dNTPs and 7 μl of diethylpyrocarbonate treated ddH_2_O (DEPC-ddH_2_O) for 5 minutes at 65°C, chilled on ice and spun quickly. This mixture was incubated with 6 μl of 5x modified RT buffer (195 mM Tris-Cl pH 8.3, 375 mM KCl), 1.5 μl of 0.1 M dithiothreitol (DTT), 40 U RNasin (Promega Corp., Australia) and 1.5 μl Superscript III for 60 minutes at 50°C. The reaction was inactivated by heating at 70°C for 15 minutes. cDNA was diluted 20-fold prior to quantitative PCR.

### Relative quantitation by real time PCR

Real-time PCR was carried out using Quantitect™ SYBR^® ^Green PCR Master Mix (Qiagen Pty Ltd, Australia) following manufacturer's instructions. Briefly, 5 μl of diluted cDNA was added to Quantitect™ SYBR^® ^Green PCR Master Mix. Forward and reverse primers were added to a final concentration of 0.3 μM. Primer sequences are listed in Table 1. A standard curve using cDNA serially diluted further to 10^-2^, 10^-4 ^and 10^-6 ^and 18S rRNA primers was created. All reactions were performed in duplicate. Real-time PCR was carried out in a Corbett Research Rotor-Gene 3000™ (Corbett Research Pty Ltd, Australia). The PCR cycling conditions included activation for 15 minutes at 95°C and 45 cycles of 30 seconds at 95°C, 30 seconds at 55°C, and 30 seconds at 72°C. Fluorescence data was recorded at the end of each 72°C step. A DNA melt profile was run subsequently from 72°C to 95°C with a ramp of 1°C/5 seconds. Fluorescence data was recorded continuously during the melt profile. The relative expression levels of EphA1, EphA2, EphB2, EphB3, EphB6, ephrin A1, ephrin A5, and ephrin B1 were calculated using the standard curve generated from the 18S rRNA gene data.

### Statistical methods

Correlation of gene expression was measured using Spearman's rho statistic. Receiver operating curve analysis was used to determine the best cut-off value for survival classification. This cut-off value was then used to analyse survival by log-rank analysis and (was visualised by) Kaplan-Meier survival curves. Hypotheses were tested using a 5% significance level. All tests were two-sided.

## Results

### Patient characteristics

Twenty-three surgical samples were obtained from operative specimens removed at the first presentation of the patient's disease and one additional sample was taken from a patient with recurrent disease. All patients underwent de-bulking surgery including total abdominal hysterectomy (TAH), bilateral salpingo-oophorectomy (BSO) and omentectomy followed by carboplatin/paclitaxel-based adjuvant chemotherapy. All patients in this study presented with stage III/IV disease with poorly differentiated serous papillary adenocarcinoma being the predominant histological type. Seven patients presented with primary peritoneal carcinoma. Median survival was 32 months with a range of 1–57 months. Individual clinical details are presented in Table 2. In this series there was no significant correlation between clinical parameters (ascites, degree of differentiation, residual tumor, tumor type or age) and survival.

### Gene expression

Initially, the expression of 20 different Eph and ephrin genes (EphA1-A8, EphB1-4 and 6, ephrin A1-5 and ephrin B1-2) were screened by quantitative real time reverse transcriptase polymerase chain reaction (Q-PCR) on two normal ovaries and ten ovarian cancer specimens. The expression levels of EphA3, A4, A5, A7, A8, B1 and B4 and of ephrin A2, A3, A4, B2 were relatively low in both normal and tumour samples and these genes were not considered further. Hence EphA1, EphA2, EphB2, EphB3, EphB6, ephrin A1, ephrin A5 and ephrin B1 were selected for further analysis in an additional fourteen tumor samples, one ovarian cyst and one ovarian adenoma. The expression levels of the two normal ovaries and two non-malignant pathological specimens were averaged and the tumor expression data are presented relative to this average (hereafter referred to as non-malignant control).

### Expression of EphA1, EphA2 and ephrin A1

**Figure 1:**
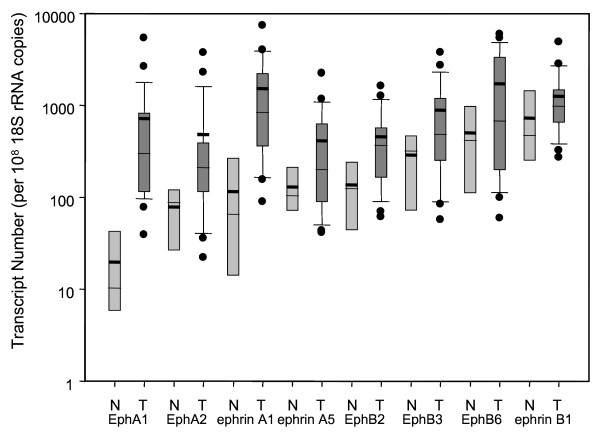
Transcript number of Eph and ephrin genes in non-malignant (N) and tumorigenic (T) tissues, relative to 10^8 ^copies of 18S rRNA shown on a log scale. Whiskers show the 10^th^–90^th ^percentile range while the shaded areas show the 25^th^–75^th ^percentile range. The thin and heavy horizontal lines represent the median and the mean respectively.

Both EphA1 and EphA2 preferentially interact with ephrin A1 and it was notable that all three were strongly expressed in a proportion of tumors. As shown in Figure 1, EphA1 was the most dramatically over-expressed gene. All tumors showed considerably higher EphA1 expression levels than those in the normal control tissues. In 22/24 tumor samples, EphA1 expression was more than five times the level seen in non-malignant controls.

EphA2 expression in all tumors was comparable to or higher than that observed in non-malignant control samples. More than half (15/23) of the tumors showed expression levels of at least two-fold greater than those seen in non-malignant control tissues, and five tumors showed a level of over-expression of greater than 5-fold.

More than half (13/24) of the tumor specimens showed greater than five-fold over-expression of ephrin A1 compared to non-malignant control samples. However, it is interesting to note that ephrin A1 correlated strongly with EphA1 (r = 0.581; p = 0.005) but only weakly with EphA2 (r = 0.387; p = 0.063).

### Expression analysis of EphB2, EphB3, EphB6 and ephrin B1

The B type Ephs and ephrins (shown in Fig 1) with higher levels of expression in this series were EphB2, EphB3, EphB6 and ephrin B1. In most samples, EphB2 expression was comparable to or only slightly higher than the average levels found in non-malignant control samples. Only three tumors showed a greater than five-fold increase in expression. A similar trend was also observed with EphB3. In these cases some tumors appeared to have reduced expression whilst others expressed higher levels including five tumors with greater than five-fold increase in expression. EphB6 was also significantly over-expressed in eleven tumors, these showing greater than five-fold over-expression compared to control tissues.

Ephrin B1 was the most highly expressed ephrin in these samples. Expression was also relatively high in non-malignant samples with no tumors showing more than two-fold over-expression. A significant correlation of expression was found between both EphB2 and EphB3 with their ligand ephrin B1 (r = 0.411; p = 0.046 and r = 0.434; p = 0.034 respectively).

### Ephrin over-expression in ovarian cancer

**Figure 2:**
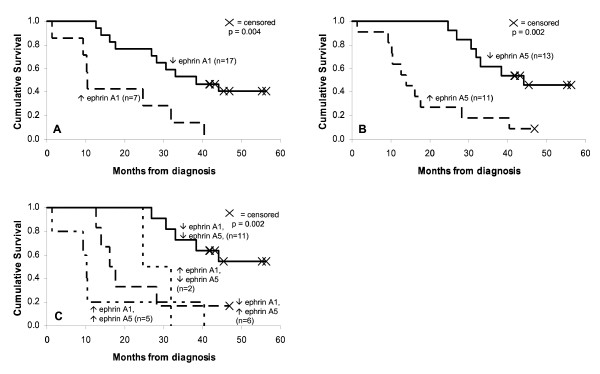
Plots showing association between high and low levels of ephrin A1 or ephrin A5, in conjunction, with survival.

Whilst neither EphA1 nor EphA2 over-expression correlated significantly with patient survival, increased expression of both ephrin A1 and ephrin A5 correlated with decreased survival. Ephrin A1, the preferential ligand for EphA1 and a high affinity ligand for EphA2, was the highest expressed ephrin in these tumors and as shown in Fig 2A, the level of expression was strongly correlated with poor survival (r = -0.470; p = 0.024). Both benign and malignant specimens showed variable ephrin A5 expression with four and eight cancer specimens demonstrating more than 5-fold and 2-fold over-expression of ephrin A5 respectively. Ephrin A5 preferentially interacts with EphA2, A3, A5, A6, A7 and B2, of which only EphA2 and EphB2 were expressed to any significant degree in the tested normal or tumor samples. Notably, ephrin A5 expression was strongly correlated with shorter overall survival (r = -0.562; p = 0.007).

Comparison of the Kaplan-Meier survival curves (Figs 2A-C) of ephrin A5 with the next best candidate gene for poor survival, ephrin A1, in conjunction with log-rank analysis, shows that ephrin A5 is the dominating influence on overall survival. Notably, survival analysis of EphB and ephrin B gene expression showed no significant correlations with survival. Survival also did not correlate with pathological categorization of tumour grade or cellularity.

## Discussion

In this study we profiled Eph and ephrin gene expression in twenty-four cases of ovarian carcinoma. Amongst the Eph proteins the striking finding was the significant over-expression of EphA1 and, to a lesser degree, EphA2 relative to the non-malignant controls. Whilst the increased proportion of epithelial cells to stromal elements in tumors explain a small increase in expression of EphA1, the very high levels of expression in a significant proportion of tumors indicates neoplastic over-expression of EphA1. We assessed cellularity of the tumours as a possible correlate, and although such analysis is limited by sampling error, we found no association of the Eph or ephrin expression levels with tumour cellularity.

Interestingly, in a proportion of these cases, there was also over-expression of ephrin A1, the preferred ligand for both EphA1 and EphA2. Notably, expression of ephrin A1 and ephrin A5, high affinity ligands for EphA2, were increased in the tumors with poorest survival. Whilst not as dramatic, there was also statistically significant co-expression of EphB2 or EphB3 with the EphB ligand, ephrin B1. The co-expression of Ephs and ephrins on the same cells suggests that tumor-tumor cell contact, could autonomously activate the Eph/ephrin system thus promoting tumour plasticity through the well described capacity for Eph/ephrin signals to induce cell-cell repulsion [1,22,23]. Whilst all cases of high ephrin expression also had high levels of Eph expression, the converse was not true. This may suggest that high Eph expression precedes ephrin over-expression, which might explain why ephrin expression is most highly correlated with tumor aggressiveness. In keeping with Eph over-expression being a priming event it is notable that EphA2 over-expression causes malignant transformation in a normal mammary epithelial cell line (MCF-10A) [19]. Interestingly, Han *et al *previously reported that EphA2 over-expression is associated with poor prognosis in ovarian cancer [24]. This did not emerge in our series, perhaps reflecting differences in sample size. Notably, both ephrin A5 and ephrin A1 are high affinity ligands for EphA2 perhaps indicating a link between the two studies.

EphA1 has also been shown to be oncogenic in the classical 3T3 fibroblast assay [25], and co-expression of an ephrin ligand could generate an autocrine loop. This has been previously described where co-expression of ephrin A1 and EphA2 was shown to have an autocrine effect [26,27].

The finding that increased expression of ephrin A5 predicted poorer prognosis was an intriguing result as this gene is generally not expressed at significant levels in epithelial tissues (unpublished observations). Ephrin A5 does not bind significantly to EphA1 [14] but has been shown to activate EphA2 in human carcinoma cells [28].

In the current study we could not correlate mRNA levels with protein expression on the biopsy samples, principally due to the lack of antibodies appropriate for immunohistochemistry. The available antibodies are generally suitable for Western blot analysis but not for immunohistochemical applications. Using the ovarian cancer lines OVCAR4 and OVCAR5 we were able to show that protein expression (Western blot analysis) correlated precisely with mRNA analysis. In the absence of suitable antibodies, future studies should include extraction of tissue lysates for Western blot analysis.

## Conclusion

Our results provide evidence of increased expression of Ephs and ephrins in ovarian cancer. This study provides evidence that ephrin, but not Eph, expression predicts poor prognosis in a group of ovarian cancer patients for whom clinical parameters were not informative. This suggests that co-expression of Eph and ephrin proteins may be a significant event in tumor progression. Whilst this study has yielded surprisingly strong correlations, a prospective study of a much larger cohort is warranted to further assess the use of ephrin expression as a useful predictor of clinical outcomes in this disease.

## Competing interests

The author(s) declare that they have no competing interests.

## Authors' contributions

NH – Participated in its design and coordination and helped to interpret and draft the manuscript, MS – Participated in its design and coordination and helped to interpret and draft the manuscript, SS – Carried out Q-PCR assays and was involved in the draft of the manuscript, TN – Extracted RNA and fixed tissue sections for histological analysis, MC – Examined all the pathological specimens, SD – Extracted RNA and was involved in the editing of the manuscript, DL – Performed statistical analyses for the manuscript, GB – Extracted RNA and fixed tissue sections for histological analysis, PP – Revisited the manuscript critically for important intellectual content, AB – Conceived the study, and participated in its design and coordination and helped to interpret and draft the manuscript.

All authors read and approved the final manuscript.

## Pre-publication history

The pre-publication history for this paper can be accessed here:



## Figures and Tables

**Table 1:** Primers used in quantitative reverse transcriptase PCR

Gene (Acc. No.)	Forward Primer (5' – 3')	Reverse Primer (5' – 3')
18S rRNA (K03432)	GACTCAACACGGGAAACCTC	AGCATGCCAGAGTCTCGTTC
EphA1 (NM_005232)	GTGGACACTGTCATAGGAGAAGG	GGTCTTAATGGCCACAGTCTTG
EphA2 (NM_004431)	GGGACCTGATGCAGAACATC	AGTTGGTGCGGAGCCAGT
ephrin A1 (BC032698)	CCGGAGAAGCTGTCTGAGAA	GGTTTGGAGATGTAGTAGTAGCTGTG
ephrin A5 (NM_001962)	TTGCACGTGGAGATGTTGAC	GGTTGCTGCTGTTCCAGTAGA
EphB2 (NM_004442)	GAAGGAGCTCAGTGAGTACAACG	GCACCTGGAAGACATAGATGG
EphB3 (NM_004443)	GGCCATAGCCTATCGGAAGT	TCCCAGTAGGGTCGCTCTC
EphB6 (NM_004445)	GAGCAGGAGGTACTAAATGCAA	CCAGCTGGTCAAAATGAGG
ephrin B1 (BC052979)	TGAAGGTTGGGCAAGATCC	GGTTCACAGTCTCATGCTTGC

Genbank Accession Numbers are listed in parenthesis.

**Table 2:** Individual clinical data for the 24 cases of ovarian cancer

**Patient Number**	ICD10*	**FIGO**^†^	**Differentiation**	**Morphology**^‡^	**Tissue Sample**	**Months to Death**	**Censored**^§^	**Residual Tissue**^||^	Ascites**
Tumor 1	C56	IIIC	Poor	PSC	Omentum	13.88	0	0	4
Tumor 2	C48.2	IIIC	Poor	PSC	Omentum	56.22	1	3	2
Tumor 3	C56	IIIC	Poor	PSC	Omentum	32.99	0	1	2
Tumor 4	C48.2	IIIC	Poor	PSC	Omentum	55.13	1	1	1
Tumor 5	C56	IIIC	Poor	PSC	Omentum	28.06	0	2	1
Tumor 6	C56	IIIC	Moderate	PSC	Ovary	44.05	0	1	2
Tumor 7	C56	IIIC	Poor	PSC	Ovary	9.24	0	2	1
Tumor 8	C56	IIIB	Poor	PSC	Ovary	38.32	0	0	1
Tumor 9	C48.2	IIIC	Poor	PSC	Omentum	40.46	0	1	1
Tumor 10	C56	IIIC	Unknown	PSC	Omentum	12.57	0	1	4
Tumor 11	C48.2	IIIC	Poor	PSC	Ovary	10.33	0	1	2
Tumor 12	C56	IIIC	Poor	PSC	Omentum	46.84	1	9	4
Tumor 13	C56	IV	Poor	PSC	Ovary	1.25	0	1	0
Tumor 14	C56	IIIC	Poor	PSC	Omentum	30.59	0	3	3
Tumor 15	C48.2	IIIC	Poor	PSC	Omentum	45.30	1	3	5
Tumor 16	C56	IIIC	Poor	PSC	Omentum	43.16	1	0	4
Tumor 17	C48.2	IIIC	Poor	SC	Omentum	26.81	0	2	4
Tumor 18	C56	IIIC	Poor	PSC	Omentum	41.81	1	0	1
Tumor 19	C56	IIIC	Poor	PSC	Omentum	41.58	1	0	2
Tumor 20	C56	IIIC	Poor	PSC	Ovary	31.78	0	0	0
Tumor 21	C56	IIIC	Poor	SC	Ovary	24.67	0	0	4
Tumor 22	C48.2	IV	Poor	PSC	Omentum	10.10	0	2	1
Tumor 23	C56	IIIC	Poor	SC	Omentum	16.09	0	3	2
Tumor 24	C56	IIIB	Poor	SC	Omentum	17.63	0	1	2

* ICD10 – International Classification of Disease v10: C48.2 – unspecified malignant neoplasm of peritoneum; and C56 – malignant neoplasm of ovary.

^†^FIGO – International Federation of Gynecology and Obstetrics Staging Classifications. Stage IIIB: involvement of both ovaries with peritoneal deposits < 2 cm; Stage IIIC: peritoneal deposits > 2 cm and/or retroperitoneal lymph node involvement; and Stage IV: involvement of both ovaries plus distant metastases.

^‡^Morphology: PSC = Papillary Serous Cystadenocarcinoma; and SC = Serous cystadenocarcinoma NOS.

^§^Censored: 0 = uncensored; and 1 = censored.

^||^Residual Tissue: 0 -nil; 1 -< 1 cm; 2 -1-2 cm; 3 – > 2 cm; and 9 – unknown.

**Ascites – 0 -nil; 1 – small (< 1L); 2 – medium (1–5L); 3 -large (> 5L); 4 – gross; and 9 – unknown
